# Greater mindful eating practice is associated with better reversal learning

**DOI:** 10.1038/s41598-018-24001-1

**Published:** 2018-04-09

**Authors:** Lieneke K. Janssen, Iris Duif, Ilke van Loon, Jeanne H. M. de Vries, Anne E. M. Speckens, Roshan Cools, Esther Aarts

**Affiliations:** 1 Radboud University, Donders Institute for Brain, Cognition and Behavior, 6500 HB Nijmegen, The Netherlands; 2 Department of Neurology, Max Planck Institute for Human Cognitive and Brain Sciences, 04303 Leipzig, Germany; 3 Integrated Research and Treatment Center (IFB) Adiposity Diseases, Leipzig University Medical Center, 04103 Leipzig, Germany; 4 Division of Human Nutrition, Wageningen University, 6700 EV Wageningen, The Netherlands; 5 Department of Psychiatry, Radboud University Medical Center, 6500 HB Nijmegen, The Netherlands

## Abstract

Mindfulness-based interventions are thought to reduce compulsive behavior such as overeating by promoting behavioral flexibility. Here the main aim was to provide support for mindfulness-mediated improvements in reversal learning, a direct measure of behavioral flexibility. We investigated whether an 8-week mindful eating intervention improved outcome-based reversal learning relative to an educational cooking (i.e., active control) intervention in a non-clinical population. Sixty-five healthy participants with a wide BMI range (19–35 kg/m^2^), who were motivated to change their eating habits, performed a deterministic reversal learning task that enabled the investigation of reward- and punishment-based reversal learning at baseline and following the intervention. No group differences in reversal learning were observed. However, time invested in the mindful eating, but not the educational cooking intervention correlated positively with changes in reversal learning, in a manner independent of outcome valence. These findings suggest that greater amount of mindfulness practice can lead to increased behavioral flexibility, which, in turn, might help overcome compulsive eating in clinical populations.

## Introduction

Mindfulness has been associated with a multitude of beneficial health outcomes^1,2^ and improvements in cognition^3^ in clinical as well as non-clinical populations. Mindfulness is defined as paying attention to present moment experience, purposefully and non-judgmentally^4^. Shapiro and colleagues^5^ have proposed that practicing mindfulness may lead to reperceiving, or *deautomization*, and suggest that one direct mechanism of action underlying the beneficial effects of mindfulness-based interventions may be increased cognitive, emotional and behavioral flexibility. Some studies have indeed shown a mindfulness-mediated increase in the ability to inhibit automatic responses, as measured with classic or emotional Stroop tasks^6–8^, which has been interpreted as evidence for increased cognitive flexibility. However, studies investigating cognitive flexibility with paradigms that require attention switching have not shown effects of mindfulness^9,10^. No studies to date have investigated the effects of mindfulness on reward-based flexibility such as reversal learning.

Reversal learning requires the ability to flexibly adapt one’s behavior in response to outcome-contingency changes in the environment and is highly relevant in the context of compulsive behavior such as addiction^11^ or overeating (i.e., consuming more energy than is expended). Although occasional overeating is a common phenomenon in our obesogenic environment, chronic overeating often results in obesity. Recent theory suggests that, rather than hyper- or hyposensitivity to food rewards, obesity may be related to impaired outcome-based learning^12–14^. Impaired reward and punishment learning may maintain overeating in obesity by leading to compulsive over-selection of actions directed at food rewards and/or decreased sensitivity to negative consequences associated with overeating, such as an uncomfortably full feeling or long-term health risks. Studies investigating cue-reward learning in relation to obesity have provided some evidence for impaired learning from positive^15,16^ as well as from negative outcomes^13,14^. Others have hypothesized that obesity is accompanied by learning impairments that generalize across outcomes^12^. Here, we hypothesize that a mindfulness-based intervention targeting undesired eating behavior would improve outcome-based reversal learning, which could be in a manner that depends on the valence of the outcome or independent of valence.

The main aim of this study was to investigate the effects of an intensive mindful eating intervention on reward- and punishment-based reversal learning. Previously, mindfulness-based interventions targeted specifically at compulsive eating behavior have been shown effective in reducing measures of overeating in healthy^17,18^, overweight and clinically obese participants^19–21^, as well as in individuals suffering from binge-eating disorder^22,23^ (but see^24^). However, only few studies have investigated the cognitive mechanisms underlying mindful eating^18,25–27^, and no study so far has focused on behavioral flexibility. To investigate the effect of mindful eating on reversal learning, sixty-five healthy human volunteers with a wide BMI range (19–35 kg/m^2^) participated in the study. They were motivated to change their undesired eating behavior (e.g. occasional overeating) and were tested before and after an intensive 8-week mindful eating intervention. The present design included a carefully matched educational cooking intervention (i.e., the active control) and participants were randomized to one of the two interventions. The use of a deterministic reversal learning task^28,29^ enabled the investigation of two separate forms of outcome-based reversal learning^30^. To contribute to the growing literature on the effectiveness of mindful eating interventions in reducing measures of overeating, the secondary aim of the study was to assess the effects of both interventions on physical measures of obesity, i.e., BMI, waist circumference and waist-to-hip ratio, as well as self-reported measures of eating behavior and knowledge of a healthy diet.

## Methods

### Participants

The results reported in this study are based on data from 65 healthy right-handed participants (55 women; mean age: 31.9 years old, SEM: 1.4, range: 19–53; mean body mass index (BMI): 26.5 kg/m^2^, SEM: 0.5, range: 19–35), and are part of a larger protocol of which other data are presented elsewhere^25,31^. Note that an earlier version of this article was previously published as part of a doctoral thesis^31^. Participants were recruited from Nijmegen and surroundings through advertisement. Only participants (aged: 18–55 years old; BMI: 19–35 kg/m^2^) with no (history of) eating disorders or current dieting, but who were highly motivated to change their undesired eating behavior such as occasional overeating, were recruited and included in the study.

Exclusion criteria were: left-handedness, inadequate demand of Dutch, current pregnancy, MRI-incompatibility, diabetes mellitus, (history of) hepatic, cardiac, respiratory, renal, cerebrovascular, endocrine, metabolic or pulmonary diseases, uncontrolled hypertension (diastolic pressure > 90 mmHg, systolic pressure > 160 mmHg), (history of) eating, neurological, or psychiatric disorders, depression/anxiety state scores > 11 on the Hospital Anxiety and Depression Scale (HADS)^32^, current strict dieting, high restrained eating score on the Dutch Eating Behavior Questionnaire (DEBQ ≥ 3.60 for males and ≥ 4.00 for females)^33^, current psychological or dietary treatment, taste or smell impairments, use of psychotropic medication, food allergies relevant to the study (pertaining to the eating exercises in the intervention programs), deafness, blindness, and sensorimotor handicaps, drug or alcohol addiction, and a change in body weight of more than 5 kg in the past two months. Crucially, participants who previously participated in an MBSR (Mindfulness-Based Stress Reduction) or MBCT (Mindfulness-Based Cognitive Therapy) course were not included in the study.

Five participants were excluded from the analyses following testing because of an incidental finding after the post-test session (n = 1), missing data (n = 1) and because of poor task performance (n = 3) (for details see Methods, Behavioral analyses).

All participants gave written informed consent and were reimbursed for the time spent in the lab, according to the local guidelines for reimbursement (i.e., 8 Euros per hour for behavioral testing, 10 Euros per hour for scanning). In addition, the intervention program was offered to the participants free of costs. The experimental protocol was approved according to institutional guidelines of the local ethics committee (CMO region Arnhem-Nijmegen, the Netherlands, 2013–188) and in accordance with the provisions of the World Medical Association Declaration of Helsinki.

### Protocol

Participants were screened for inclusion and exclusion criteria, and matching criteria (age, gender, BMI, experience with meditation and yoga) were assessed by taking physical measures and administering self-report questionnaires on a separate intake session. To assess verbal intelligence and education level, the Dutch version of the National Adult Reading Test was administered^34^. Experience with meditation and yoga was assessed by means of an in-house self-report questionnaire.

After inclusion, participants came to the laboratory in the month before and after the intervention, referred to as pre- and post-test session. Test sessions started at 11:00 AM or 12:30 PM, which was kept constant within participants as much as possible. Participants were instructed to abstain from drinking alcohol 24 hours before the test session. Before testing, physical measurements of obesity were taken (weight, waist and hip circumference), digit span was assessed^35^, and self-report questionnaires were administered to characterize the participants (Table 1). The following self-report questionnaires and scales were administered for explanatory purposes (i.e., to characterize the sample and account for between-group differences at baseline that could occur by chance), and to address the secondary aim of the study (i.e., to assess the effectiveness of the intervention programs in physical and self-reported measures of overeating) (Table 2): the Fagerstrom Test for Nicotine Dependence (FTND)^36^ to assess smoking and nicotine dependence; the Positive And Negative Affect Scale (PANAS)^37^ to assess positive and negative affect before scanning; the Barratt Impulsiveness Scale-11 (BIS-11)^38^ to assess impulsivity; the Behavioral Inhibition System/Behavioral Approach System questionnaire (BIS/BAS)^39^ to assess punishment and reward sensitivity; the Kirby questionnaire^40^ to assess delayed reward discounting; the Food Frequency Questionnaire, Dutch Healthy Diet (FFQ-DHD)^41^ to assess the degree to which participants eat according to the national guidelines for a Dutch healthy diet; a shortened version of the Food Behavior Questionnaire (FBQ) to assess behavior towards food; the Dutch Eating Behaviour Questionnaire (DEBQ)^42^ to assess emotional, external and restraint eating behavior; the Five Facet Mindfulness Questionnaire – Short Form (FFMQ-SF)^43^ to assess degree of mindfulness; the Hospital Anxiety and Depression Scale (HADS)^32^ to assess levels of anxiety and depression; a Treatment Credibility Questionnaire (TCQ) to assess how much participants believed the intervention would work for them. Note that the pre-measurement of the TCQ was taken at the first session of the intervention program rather than on the pre-test session, as participants were unaware of the contents of their intervention at that time. Prior to the reversal learning task reported here, participants underwent a one hour MR scanning session, in which they performed a food Stroop task^44^ and a monetary and caloric incentive delay task^25^, followed by an outcome devaluation task outside the scanner^44^. The results from these tasks are reported elsewhere as the tasks addressed different research questions.

**Table 1:** Between-group comparisons.

	Mindful eating (n = 35)			Educational cooking (n = 30)			*p*	test-statistic^a^
Gender (Male:Female)	5:30			5:25			*1.000*	*na* ^b^
Age (yrs)	32.3	±1.9	20–52	31.3	±2.2	19–53	*0.717*	*0.364*
Body mass index (kg/m^2^)	26.7	±0.7	19–35	26.3	±0.7	20–35	*0.635*	*0.476*
Yoga/meditation experience (yrs)	2.2	±0.8	0–21	3.8	±1.2	0–31	*0.271*	*−1.110*
Education (NART)	6.5	±0.1	5–7	6.3	±0.1	5–7	*0.076*	*405.0* ^c^
Verbal IQ (NART)	107.9	±1.8	90–132	102.4	±1.5	88–123	*0.036*	*366.5* ^c^
Working memory (digit span total)	15.7	±0.6	9–23	14.3	±0.6	9–22	*0.110*	*1.622*
Smoking (FTND)	0	±0	0	0.03	±0.03	0–1	*0.280*	*507.5* ^c^
Time on training (hrs)	29.8	±2.6	0–51	33.4	±3.5	3–78	*0.404*	*−0.840*
Attendance (^#^of sessions)	6.2	±0.4	1–9	6.5	±0.5	1–9	*0.582*	*484.0* ^c^
Attendance < 4 sessions (n)	7			5			*0.761*	*na* ^b^

If not otherwise stated, values represent mean ±SEM, and min-max. *NART*: National Adult Reading Test (in Dutch (NLV)) was administered to assess education, which is measured on a scale of 1–7 (no degree – academic degree); *FTND*: Fagerstrom Test for Nicotine Dependence.

^a^If not otherwise stated: independent samples-tests (degrees of freedom: 63).

^b^Based on Fisher’s Exact Test; ^c^Mann-Whitney U test.

**Table 2:** Means and standard errors of the mean of pre and post intervention measurements for each group, and Time (pre, post) × Intervention (ME, EC) statistics.

	Mindful eating (n = 35)				Educational cooking (n = 30)				*p*	test-statistic^a^
	pre		post		pre		post			
**Physical measurements**										
BMI (kg/m^2^)	26.7	±0.7	26.9	±0.7	26.3	±0.7	25.9	±0.7	***0.005***	*8.634*
Waist (in cm)	90.3	±2.3	90.2	±2.4	87.9	±2.3	86.0	±2.4	***0.021***	*5.565*
Waist-to-hip ratio	0.85	±0.01	0.84	±0.01	0.84	±0.01	0.84	±0.01	*0.738*	*0.113*
**Self-report questionnaires**										
BIS	21.0	±0.5	20.5	±0.5	20.0	±0.6	19.5	±0.6	*0.978*	*0.001*
BAS	41.8	±0.6	42.2	±0.7	43.7	±0.7	43.4	±0.8	*0.370*	*0.814*
FFQ – DHD	52.9	±1.8	54.5	±1.7	48.9	±2.5	58.8	±2.1	***0.005***	*8.670*
**FBQ – short version**										
Knowledge	15.6	±0.2	15.9	±0.2	14.8	±0.3	16.7	±0.1	<***0.001***	*23.522*
Temptation	27.9	±1.0	27.9	±0.9	27.8	±0.7	26.7	±0.7	*0.241*	*1.398*
**DEBQ**										
Restraint	2.5	±0.2	2.9	±0.1	2.7	±0.1	3.0	±0.1	*0.693*	*0.157*
Emotional	2.6	±0.2	2.7	±0.1	2.9	±0.2	2.7	±0.2	*0.218*	*1.551*
External	2.8	±0.2	3.1	±0.1	3.2	±0.1	3.2	±0.1	*0.116*	*2.544*
FFMQ-SF^c^	77.3	±1.5	75.1	±1.3	76.4	±1.5	76.6	±1.5	*0.517*	*0.426*
**HADS**										
Anxiety	3.9	±0.5	6.0	±0.4	5.3	±0.6	5.9	±0.7	*0.050*	*3.977*
Depression	2.1	±0.4	2.7	±0.4	2.7	±0.5	2.8	±0.5	*0.281*	*1.180*
TCQ^d^	26.9	±2.0	27.0	±1.5	32.2	±0.9	32.4	±1.5	*0.977*	*0.001*

If not otherwise stated, values represent mean ±SEM.

*BIS/BAS*: Behavioral Inhibition System/Behavioral Approach System questionnaire; *FFQ-DHD*: Food Frequency Questionnaire, Dutch Healthy Diet; *FBQ*: Food Behavior Questionnaire, a shortened version; *DEBQ*: Dutch Eating Behaviour Questionnaire; *FFMQ-SF*: Five Facet Mindfulness Questionnaire – Short Form; *HADS*: Hospital Anxiety and Depression Scale; *TCQ*: Treatment Credibility Questionnaire. Note that the pre-TCQ was filled out at the first session of the intervention, not on the pre test session, as participants were unaware of the contents of the assigned intervention at that time.

^a^If not otherwise stated, the reported test-statistic is the F-value (degrees of freedom: 1,63).

^b^Mann-Whitney U.

^c^FFMQ-SF: N = 54 (N_ME_ = 24, N_EC_ = 30; degrees of freedom: 1,52).

^d^TCQ: N = 55 (N_ME_ = 29, N_EC_ = 26; degrees of freedom: 1,53).

One year after the intervention, participants were invited back to the laboratory to reassess physical measurements of obesity (weight, waist and hip circumference) and the self-report questionnaires as administered on pre- and post-test sessions. Reversal learning was not reassessed at one-year follow-up.

### Paradigm

**Figure 1:**
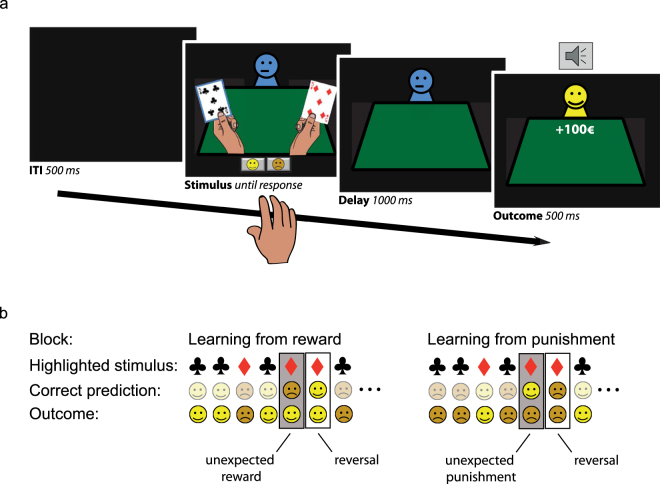
Reversal learning task as was previously used in Janssen *et al*.^28^. (**a**) On each trial, participants were presented with 2 gambling cards. One of the cards was selected by the computer and highlighted with a blue border. Participants then had to predict, with a left or right button press, whether the card would be followed by a reward (a happy emoticon, +100€ sign, and a high-pitch tone) or punishment (a sad emoticon, −100€ sign, and a low-pitch tone). After a short delay, the outcome was presented. (**b**) An example of a sequence of trials until reversal for both the learning from reward (left) and punishment block (right). The participant learned to predict reward and punishment for the two gambling cards. The card-outcome associations were deterministic, and reversed after 5 to 9 correct responses. Reversals were signaled by either unexpected reward (reward block) or unexpected punishment (punishment block). Performance was measured on reversal trials, immediately after the unexpected outcomes.

Participants performed a version of the deterministic reversal learning task^28^ adapted from Cools *et al*.^29,45^. The task was programmed with Presentation software (Version 16, Neurobiobehavioral Systems, Inc.). On each trial, participants were presented with two playing cards simultaneously, one of which was highlighted (Fig. 1). One of the two cards was associated with upcoming reward, the other one with upcoming punishment. Participants had to learn to predict the outcome associated with this preselected card by trial-and-error. Responses were self-paced and were made by pressing one of two buttons associated with either reward or punishment using the right index or middle finger (counterbalanced across participants). After a short delay (1000 ms) the outcome was presented (500 ms). Note that the outcomes were not contingent on the participants’ responses, but on the highlighted stimulus; thus, contingencies were Pavlovian rather than instrumental. The stimulus-outcome contingency reversed after five to nine consecutive correct predictions. Participants performed two blocks, each consisting of 240 trials (i.e. a total of 480 trials). In one block, reversals were always signaled by unexpected rewards (smiling emoticon with a “+€100” sign and a high-pitch tone), and in the other block reversals were always signaled by unexpected punishments (sad emoticon with a “−€100” sign and a low-pitch tone). The order of blocks was counterbalanced between sessions and across participants. Error rate on the trials immediately after reversals (i.e. unexpected reward or punishment) indexes the ability to update predictions of reward and punishment, i.e., how well participants learned from either unexpected reward or unexpected punishment. Participants were instructed according to the original procedure by Cools *et al*.^29^ and were trained extensively before the experiment so that they understood the structure of the task and the Pavlovian, instead of instrumental nature of the contingencies.

The task enabled the investigation of two separate forms of outcome-based reversal learning^30^. First, participants were required to learn Pavlovian associations between stimuli and their outcomes (i.e., reward or punishment). The ability to learn from positive and negative prediction errors, i.e. unexpected rewards and punishments, was quantified in terms of valence-dependent reversal learning (error rate unexpected reward > unexpected punishment). Second, averaging learning across reward and punishment in this task, i.e., valence-independent reversal learning, provides a measure of behavioral flexibility in general.

### Interventions

Participants were randomly assigned to one of two intervention programs that were offered free of costs: mindful eating (ME) or educational cooking (EC; active control), using minimization^46^ with respect to age (categories: 18–25, 26–35, 36–45, 46–55 years old), gender (categories: male, female), BMI (categories: 19–24.9, 25–29.9, 30–35 kg/m^2^) and experience with meditation and yoga (categories: never, 0–2, 2–5, 5–10, >10 years). Randomization was computer-based and relied on an algorithm that assigned participants to one of the groups by taking into account the given minimization factors, which guaranteed that the groups were balanced in terms of these factors. The algorithm with study-specific minimization factors was implemented by an independent statistician from Radboud University Medical Center.

**Figure 2:**
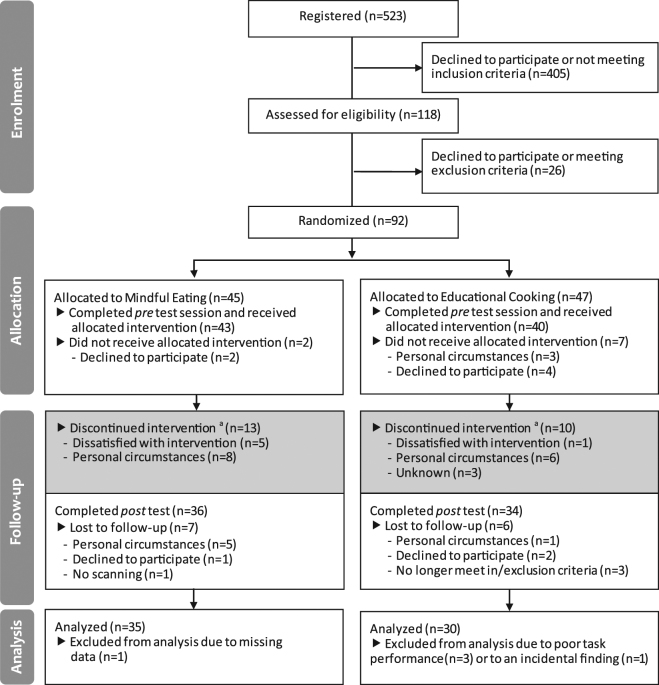
CONSORT flow diagram. ^a^Attended <4 sessions of the intervention program. Note that these participants were invited back to the laboratory for the *post* test session.

The intervention programs were matched in terms of time, effort, and group contact, but differed significantly in terms of content. Both programs consisted of 8 weekly, 2.5 hour group sessions from 7 PM–9:30 PM, plus one day dedicated (6 hours) to the intervention goals. Participants were asked to spend 45 minutes per day on homework assignments and to record the amount of time spent on homework forms. Participants were encouraged to complete as much of the homework as possible, but more importantly, to accurately report on their actual time-investment to prevent dishonest reporting. The intervention programs were described as “eating with attention” (ME) and “eating with knowledge” (EC) to prevent a selection-bias of participants interested in mindfulness. Only after the first test session, participants were informed about the intervention to which they were randomized, to ensure that baseline measurements were not influenced by intervention expectations. Because group size was set to 10 to 15 participants per round, included participants were divided across three rounds for each intervention (3 × ME, 3 × EC). The final sample for the analyses reported here consisted of 35 participants in the ME intervention and 30 participants in the EC intervention (for a flow diagram see Fig. 2).

#### Mindful eating (ME)

The aim of the ME intervention was to increase experiential awareness of food and eating (e.g. being more aware of food taste and smell, thoughts and feelings during eating or cravings, and internal signals like satiety). The program was based on the original MBSR program developed by Kabat-Zinn *et al*.^47^ at the Stress Reduction and Relaxation Clinic, Massachusetts Medical Center. Participants performed formal mindfulness practices (i.e. body scan, sitting meditation, walking meditation and mindful movement), aimed at increasing general mindfulness skills, which were similar to the original program. In addition, participants performed informal mindfulness practices based on the Mindful Eating, Conscious Living program (MECL)^48^, which were mainly directed to mindful eating and not part of the original MBSR program. Sessions focused on themes, such as: the automatic pilot, perception of hunger and other internal states, creating awareness for boundaries in eating behavior, stress-related eating, coping with stress, coping with (negative) thoughts, self-compassion, and how to incorporate mindfulness in daily life. Towards the end of the program, participants had a silent day. During this day, the whole group performed formal mindfulness exercises and ate a meal together in complete silence. Homework consisted of a formal mindfulness practice, using CDs with guided mindfulness exercises, and an informal mindfulness practice directed at one moment (e.g. a meal) a day. Time spent on homework was noted on homework forms every day. The ME intervention was developed and delivered by qualified psychologists/psychiatrists, who graduated from the post-graduate mindfulness teacher training at the Radboud University Medical Center for Mindfulness.

#### Educational Cooking (EC)

The aim of the EC intervention was to increase informational awareness of food and eating. The program was based on the Dutch healthy diet guidelines (www.voedingscentrum.nl). To establish similar group contact and activities (vs. passive listening) as in the ME, participants were actively enrolled in cooking workshops during the group meetings of the EC. Sessions focused on healthy eating, healthy cooking of vegetables and fruit, use of different types of fat and salt for cooking, reading of nutrition labels on food products, healthy snacking, guidelines for making healthy choices when eating in restaurants, and how to incorporate healthy eating and cooking in daily life. Towards the end of the program, participants had a balance day, during which the participants adhered to all nutritional health guidelines for every snack and meal. Homework assignments entailed practicing cooking techniques, or grocery shopping with informational awareness (i.e. reading food labels for nutritional content), and counting the amount of calorie intake for one meal a day (to be noted in a homework diary). The EC intervention was developed and delivered by a qualified dietitian from Wageningen University and the cooking sessions were guided by a professional chef. Sessions took place at a large kitchen facility of the Nutrition and Dietetics faculty of the Hogeschool of Arnhem-Nijmegen.

### Analyses

Overall error rates (ER) and error rates on reversal trials (trials immediately following unexpected outcomes) were arcsine transformed as is appropriate when variance is proportional to the mean^49^. To investigate performance in general the transformed overall error rates were analyzed using a mixed ANOVA (SPSS 19, Chicago, IL) with Time (pre vs. post) as within-subject factor and Intervention (ME vs. EC) as a between-subject factor. Transformed error rates on reversal trials were analyzed using the same design, but with Valence (unexpected reward vs. punishment) as an extra within-subject factor.

**Figure 3:**
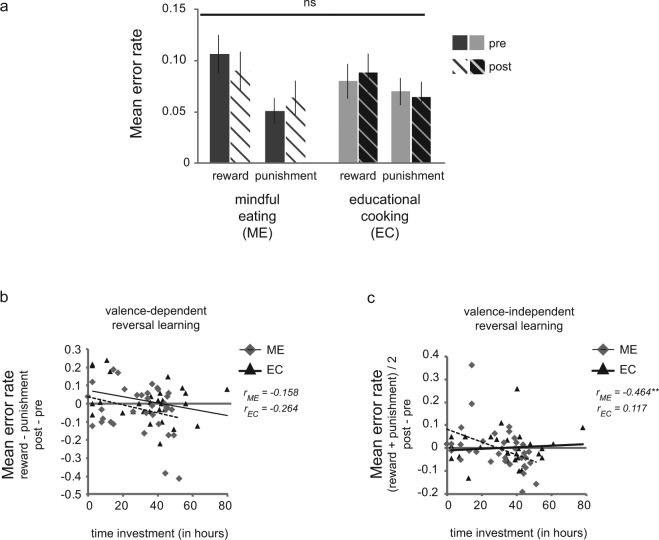
Intervention effects on reversal learning. (**a**) The mindful eating (ME) and educational cooking (EC; active control) intervention did not differentially affect reversal learning from reward or punishment. Overall participants were impaired at learning from reward relative to punishment (main effect Valence: F(1,63) = 10.1, p = 0.002, η_p_^2^ = 0.138). (**b**) Valence-dependent reversal learning (i.e. mean error rates on trials following reversals signaled by unexpected reward - unexpected punishment) was not associated with the amount of time participants invested in either intervention (p’s>0.15). (**c**) Better valence-independent reversal learning (i.e. *lower* mean error rates on trials following reversals in general ([unexpected reward + unexpected punishment]/2)) was related to increased time investment in the mindful eating (dashed line: r = −0.464, p = 0.005), but not the educational cooking training (solid line: r = 0.117, p = 0.538). Note that whereas the statistics were performed on the transformed error rates (see Methods), the untransformed error rates were plotted for illustrative purposes. Error bars reflect 1 SEM.

Valence-dependent and valence-independent reversal learning scores were calculated by computing, respectively, the difference between, and the average of the error rates on reward and punishment reversal trials (see Fig. 3a,c). Effects of mindfulness-based interventions have been shown to depend on the amount of time individuals have spent on it^50^. Therefore, we ran *post hoc* correlational analysis using Pearson’s r to investigate the change in both valence-dependent as well as valence-independent reversal learning scores (post – pre) as a function of time investment for both interventions, and statistically compared the slopes between the ME and EC group using Fisher’s r-to-z transformation. The significance level was Bonferroni-corrected for the two comparisons and set to α = 0.025.

In addition, the total number of reversals obtained throughout the task were analyzed using the same mixed design. Because the stimulus-outcome contingency in the task reversed after five to nine consecutive correct predictions, and the total number of trials was fixed, the number of reversals for the reward and punishment block reflects performance also on the non-reversal trials. Three participants performed poorly on the task in terms of overall error rate (n = 1, ER>0.7) or number of reversals (n = 2, <10 reversals per condition) and were excluded from the task based on Grubbs’ test for outliers^51^.

Finally, we analyzed secondary outcome measures related to food intake. Between-group comparisons in self-report measures were analyzed using independent-samples t-tests, Fisher’s Exact Tests, or Mann-Whitney U tests (Table 1). Effects of Time (pre vs. post) and Intervention (ME vs. EC) on physical as well as self-report measures were analyzed using ANOVAs, or Mann-Whitney U tests (Table 2). To assess the longevity of measures exhibiting a significant Time x Intervention interaction, we ran *post hoc* ANOVAs adding the one-year follow-up data as a third level in factor Time for BMI, waist, FFQ-DHD and FBQ knowledge. Data was available for 27 participants in the ME group and 24 participants in the EC group (n_EC_ = 23 for self-report measures due to missing values). In case of violation of the assumption of sphericity as indicated by Mauchly’s test, Greenhouse-Geisser correction was used to adjust the degrees of freedom accordingly (see Results). Planned *post hoc* comparisons were performed to statistically compare follow-up data to data from both the pre and post test sessions separately.

### Data availability

The dataset generated and analyzed during the current study are available from the corresponding author upon reasonable request.

## Results

ANOVA of error rates on reversal trials (i.e. trials immediately following reversals) revealed that participants made more errors after unexpected reward compared with unexpected punishment (main Valence: F(1,63) = 10.1, p = 0.002, η_p_^2^ = 0.138) (Fig. 3a). The analysis showed no differential effect of the interventions on valence-independent reversal learning (interaction Time × Intervention: F(1,63) = 0.044, p = 0.835, η_p_^2^ = 0.001) nor on valence-dependent reversal learning (interaction Valence × Time × Intervention: F(1,63) = 1.7, p = 0.200, η_p_^2^ = 0.026). These findings suggest that overall the mindful eating (ME) intervention did not differentially affect reversal learning relative to educational cooking (EC).

However, we observed large individual differences between participants in terms of total time invested in the intervention program (i.e., time on attended sessions and homework), which did not significantly differ between the groups (Table 1). One might expect that effects of the mindfulness intervention on reversal learning depend on total time invested. *Post hoc* correlational analyses for change (post – pre) in both valence-dependent and valence-independent reversal learning addressed this hypothesis. Time investment and reduced error rates for valence-independent reversal learning correlated significantly in the ME group (r = −0.464, p = 0.005), but not in the EC group (r = 0.117, p = 0.538) (Fig. 3). The correlation coefficients differed significantly between groups (Fisher’s z = −2.37, p = 0.018). This suggests that participants who invested more time on the mindful eating intervention improved more in terms of valence-independent reversal learning, whereas no such improvement was observed for participants in the active control group. We did not find intervention effects on valence-dependent reversal learning as a function of time-investment (all p’s < 0.15).

The reported findings cannot be explained by group differences in overall performance between the ME and EC groups given the absence of main or interaction effects of Intervention on error rates across trials, i.e. reversal and non-reversal trials (F(1,63)’s < 1, p’s > 0.5), nor did overall performance correlate with time investment (all p’s > 0.4). In addition, ANOVA of the number of reversals revealed no interaction effect of Valence x Time x Intervention (F(1,63) = 2.603, p = 0.112), or other Intervention effects (F(1,63)’s < 1, p > 0.5).

Furthermore, the groups were well matched in terms of the minimization factors age, gender, BMI and experience with yoga and meditation (Table 1). However, the ME group scored significantly higher in terms of verbal IQ (Mann-Whitney U = 366.5, p = 0.036) and was marginally more highly educated than the EC group (Mann-Whitney U = 405.0, p = 0.076)(Table 1). *Post hoc* non-parametric analyses revealed no correlation between change in valence-independent reversal learning score (post-pre) and education (r_s_ = −0.093, p = 0.461), but a significant correlation with verbal IQ (r_s_-0.313, p = 0.011). Verbal IQ did not significantly correlate with time invested in the intervention either within or across the groups (p’s > 0.1). Importantly, multiple regression analysis of valence-independent error rates following mindful eating including time invested and verbal IQ as regressors (as well as baseline valence-independent error rate) showed that time investment significantly predicted error rates (β_standardized_ = −0.397, p = 0.014), whereas verbal IQ did not (β_standardized_ = −0.117, p = 0.490)(baseline valence-independent error rate: β_standardized_ = 0.516, p = 0.005). This suggests that verbal IQ cannot explain the observed findings.

Next, the secondary outcome measures related to food intake were assessed. As observed in a largely overlapping sample^25^, the interventions differentially affected physical measures of obesity as indicated by a significant Time × Intervention interaction (Table 2). BMI and waist were decreased following educational cooking (EC; main Time: BMI: F(1,29) = 6.7, p = 0.015, η_p_^2^ = 0.188; waist circumference: F(1,29) = 13.4, p = 0.001, η_p_^2^ = 0.315), but not following mindful eating (ME; main Time: BMI: F(1,34) = 2.0, p = 0.166, η_p_^2^ = 0.056; waist circumference: F(1,34) < 1, p = 0.881, η_p_^2^ = 0.002). Across the intervention groups, greater reductions in BMI were associated with greater time spent on the intervention (r = −0.286, p = 0.021), although this was not significant for either group (ME: r = −0.252, p = 0.145; EC: r = −0.280, p = 0.134). Furthermore, EC participants reported closer compliance to the Dutch guidelines for healthy eating (main Time: F(1,29) = 17.3, p < 0.001, η_p_^2^ = 0.373) than ME participants (main Time: F(1,34) = 1.1, p = 0.311, η_p_^2^ = 0.030) as substantiated by a significant Time × Intervention interaction for FFQ-DHD scores (Table 2). EC participants also showed a significant increase in knowledge on healthy eating following the intervention (main Time: F(1,29) = 59.6, p < 0.001, η_p_^2^ = 0.673), whereas ME participants did not (main Time: F(1,34) < 1, p = 0.346, η_p_^2^ = 0.026) as evidenced by a significant Time × Intervention interaction for FBQ scores (Table 2). Analyses of the other self-report questionnaires revealed no significant interactions between Time and Intervention.

**Figure 4:**
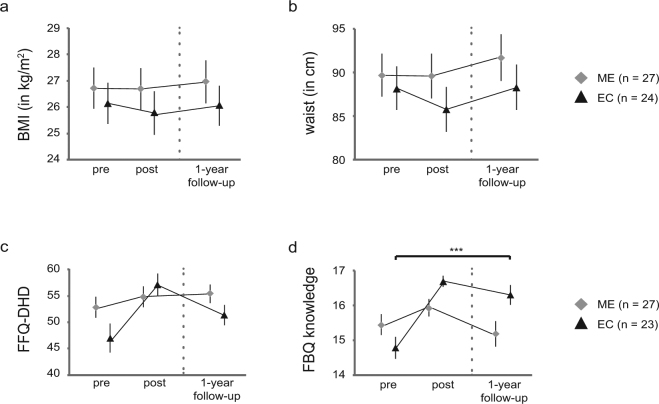
Long-term intervention effects on secondary outcome measures. Mean BMI (**a**), waist circumference (**b**), and self-reported compliance to the Dutch guidelines for healthy eating (FFQ-DHD; (**c**) did not significantly differ from pre test session to one-year follow-up, whereas knowledge on healthy eating (FBQ; (**d**) remained significantly higher for the EC group at one-year follow-up (t(22) = −4.288, p < 0.001). Error bars reflect 1 SEM.

Finally, to establish whether the observed interactions between Time and Intervention in physical and self-report measures were long-lasting, we ran *post hoc* analyses by adding the one-year follow-up data as an extra level of factor Time (pre, post, follow-up) in the ANOVAs for all participants from the reported sample that returned for the follow-up. As indicated by Mauchly’s test, the assumption of sphericity was violated for BMI (χ^2^(2) = 0.6, p < 0.001) and waist (χ^2^(2) = 0.6, p < 0.001). Degrees of freedom were therefore corrected using Greenhouse-Geisser estimates of sphericity (ε_BMI_ = 0.727; ε_waist_ = 0.720). For BMI and waist the Intervention × Time interaction was no longer significant (BMI: F(1.454,71.224) = 1.0, p = 0.350, η_p_^2^ = 0.020; waist: F(1.439,70.524) = 1.713, p = 0.194, η_p_^2^ = 0.034). No main effects of either Time or Intervention (all p’s > 0.1) were observed. The interactions for both self-reported compliance to the Dutch guidelines for healthy diet (FFQ-DHD) and knowledge on healthy eating (FBQ) remained significant (FFQ-DHD: F(2,47) = 3.8, p = 0.029, η_p_^2^ = 0.140; FBQ: F(2,47) = 8.9, p = 0.001, η_p_^2^ = 0.274). Planned *post hoc* comparisons revealed that there were no significant differences between pre measurements and one-year follow-up measurements for BMI, waist and self-reported compliance to the Dutch guidelines for a healthy diet for either group, whereas knowledge on healthy eating differed still differed significantly for EC participants at one-year follow-up compared to pre measurements (Fig. 4).

## Discussion

In this actively controlled study, we investigated the effects of an 8-week mindful eating (ME) intervention on reward- and punishment-based reversal learning using a deterministic stimulus-outcome reversal learning task. No overall group differences were found for either form of reversal learning following the interventions. However, relative to the educational cooking (EC; i.e. active control) group, the mindful eating (ME) group showed changes in valence-independent reversal learning depending on the time participants invested in the program. More specifically, participants who invested more time in the mindful eating intervention improved in valence-independent reversal learning, suggesting increased behavioral flexibility after practicing mindful eating. The effect did not vary as a function of the valence of the outcome signaling the need for reversal.

This is the first study investigating the effects of an intensive mindfulness-based intervention on the ability to adjust behavior following unexpected outcomes. The observed mindfulness-mediated increase in behavioral flexibility is in line with the cognitive, emotional and behavioral flexibility theory by Shapiro and colleagues^5^ on the mechanisms underlying the beneficial effects of mindfulness. Flexibility requires the ability to switch and adapt a strategy to face unexpected conditions^6^. Oberle and colleagues^52^ have indeed found inhibitory control in a task-switching paradigm to correlate positively with self-reported disposition of mindfulness in adolescents. Furthermore, previous studies have shown mindfulness-mediated increases in the ability to inhibit prepotent responses during response conflict as measured using classic or emotional Stroop tasks^6–8^. It has been argued that the response conflict effect is smaller for those individuals who can more flexibly disengage from highly automatized responses^6^. A decrease of the Stroop conflict effect was found for experienced meditators relative to meditation-naive controls^6^, as well as following a brief mindfulness meditation in meditation-naive individuals^7^ and in individuals with generalized anxiety disorder^8^. In contrast, several studies tapping into cognitive flexibility more directly by investigating improvement in attention switching have shown no effect of a 10-day mindfulness meditation retreat^9^ or an 8-week mindfulness-based stress reduction course^10^ relative to a passive control group. This generally concurs with the absence of an overall group effect on valence-independent reversal learning in the current study. Although reversal learning paradigms tap into behavioral rather than cognitive flexibility, it is possible that effects of mindfulness on attention switching in previous studies would have surfaced as a function of time invested in the interventions. This hypothesis awaits confirmation by future studies.

Previous work has reported reduced impulsive decision making in the food, but not monetary domain following a brief mindful eating workshop relative to an active control group, independently of BMI^26^. Accordingly, it is possible that a group effect on this version (not food-related) of the reversal paradigm could have been observed when using food rather than non-food outcomes. Note that the mindfulness-based intervention in the current study was specifically targeted at reducing bad eating habits, which may particularly reduce saliency for food rewards and have less of an effect on other, less problematic, reward domains. Affective saliency in general, unrelated to the problematic reward domain, might only be targeted by more advanced stages of mindfulness practice. This was suggested by Allen and colleagues^50^, who found improvements in affective processing in a number-counting Stroop task with interfering negatively valenced pictures, only in those individuals who invested the most time in mindfulness practice, whereas cognitive control improved in the mindfulness group relative to the active control group, independent of time investment. This may also explain why Kirk and Montague^53^ have found a relationship between mindfulness and neural measures of Pavlovian prediction errors in experienced meditators relative to naive controls, while we do not find mindfulness-mediated changes in reversal learning following Pavlovian (i.e., valence-dependent) prediction errors in individuals without previous meditation-experience. We speculate that improvements in valence-dependent reversal learning might be observed in more experienced meditators, who have invested considerable amounts of time on their meditation practice. Future research is required to confirm this hypothesis.

As in a largely overlapping sample^25^, we did not find evidence of altered eating behavior following the mindful eating intervention as would be evidenced by a decrease in secondary outcome measures, i.e. BMI, waist, WHR, or self-report measures of external or emotional eating, or of eating according to the guidelines for a Dutch healthy diet. This contrasts with several other studies, reporting reduced measures of overeating, such as consumption of sweets^54^, binges, externally and emotionally driven eating^21^ and BMI^20^ in non-clinical populations, as well as of number of binges in binge-eating disorder^22^. A possible explanation for the absence of reduced physical measures of obesity following mindful eating is that mindfulness has been found to decrease body image concern in healthy women with disordered eating behavior^21^. Decreased body image concern may have reduced the explicit motivation to lose weight despite being healthy in part of the participants in this study. Future studies should take into account body image concern to confirm or rule out this possibility. Furthermore, the current sample was rather heterogeneous in terms of the motivation to take part in the intervention, which may be reflected in a lower mean BMI compared with previous studies in healthy populations^20,21^. Previous studies showing beneficial effects of mindful eating on measures of overeating as mentioned above, investigated more homogeneous groups of participants, for example, individuals suffering from binge-eating disorder^22^ or specifically healthy women reporting problematic eating behavior^21^. As a consequence of the heterogeneity as well as the limited sample size, the study might have been underpowered to reveal effects of mindfulness on secondary outcome measures. However, note that in general the evidence on the effectiveness of mindfulness for specifically weight loss is relatively scarce despite larger sample sizes^55,56^, in particular when it comes to long-term maintenance^57^. In fact, the few studies that have shown such an effect of mindfulness on weight loss were those with a focus on weight loss (as reviewed by^56^), in contrast to the aim of the mindful eating intervention employed in the current study. Note that a reduction in physical measures (i.e., BMI and waist circumference) and an increase in adherence to the guidelines for a Dutch healthy diet were in fact observed in the educational cooking group. This is not surprising given the aim of the educational cooking intervention, i.e., following a healthier eating pattern. As part of the homework assignments, participants were instructed to adhere to the guidelines for a Dutch healthy diet, with reduced intake of sugar, fats and salt. This is likely to result in reduced BMI and waist circumference, as well as in increased adherence to these guidelines and increased knowledge thereof.

Given the reported health benefits of the educational cooking intervention in this study and the cognitive improvement with significant amounts of mindful eating practice, it might be fruitful to develop a combined program. Although weight control and diet interventions are often successful in producing significant weight loss, they often fail to produce long-term weight maintenance^58^. This is supported by the *post hoc* analyses of BMI, waist circumference, and self-reported compliance to the Dutch healthy diet guidelines (FFQ-DHD) at one-year follow-up in the present study, showing that these measures returned to baseline one year after the intervention, despite the fact that knowledge of healthy eating remained significantly higher compared to baseline in the EC group. Previous studies investigating factors contributing to successful weight maintenance have shown that behavioral flexibility is beneficial, i.e., individuals with more flexible styles of behavioral adjustment in terms of, for example, responding to cravings were more likely to maintain a healthy weight or to not become overweight^59,60^. We speculate that a combination of the two interventions in this study might therefore lead to health benefits that are more easily maintained due to increased behavioral flexibility.

However, mindfulness might also contribute to successful weight maintenance by acting on different mechanisms than behavioral flexibility. For example, stress reduction^2^ and increased self-compassion (being compassionate with oneself)^57,61^ are important aspects of mindfulness-based intervention programs and have been associated with successful behavioral change in health contexts. Although both self-compassion and stress reduction were integral parts of the present mindful eating program, our study design does not allow for a systematic investigation of the effects of mindful eating on these mechanisms. Future studies investigating a combined intervention of mindful eating and informational awareness of healthy eating, could address whether the success of behavioral change is mediated by behavioral flexibility, reduced stress or increased self-compassion, and whether or not the mechanism of action differs between individuals.

The gender imbalance in the current study limits our interpretation. Since the majority of the sample consisted of women, statistical power was not sufficiently strong to show that the observed results generalize to men.

In short, this study shows that time invested in an intensive 8-week mindful eating intervention by individuals struggling with their eating habits is associated with improvements in reversal learning. A mindfulness-mediated increase in behavioral flexibility may help overcome undesired eating habits. Future longitudinal and actively controlled studies are needed to confirm the hypothesis that a mindfulness-mediated increase in behavioral flexibility might result in long-term impact of interventions aimed at changing eating habits such as weight control programs, and to assess in more detail the mechanism by which this works.
